# Evolution of Delusional Disorder across the DSM editions

**DOI:** 10.1192/j.eurpsy.2022.1143

**Published:** 2022-09-01

**Authors:** M. Conde Moreno, F. Ramalheira, D. Vila-Chã, D. Terêncio

**Affiliations:** 1 Centro hospitalar Psiquiátrico de Lisboa, Hospital De Dia, Lisboa, Portugal; 2 Centro hospitalar Psiquiátrico de Lisboa, Serviço De Electroconvulsoterapia, Lisboa, Portugal; 3 Centro Hospitalar Psiquiátrico de Lisboa, Clínica 1, Lisboa, Portugal; 4 Centro Hospitalar Psiquiátrico de Lisboa, Psiquiatria, Lisboa, Portugal

**Keywords:** Delusional disorder

## Abstract

**Introduction:**

States compatible with “Delusional disorder” have been described since the XIX century. Esquirol mentioned “irrational ideas and actions that would develop via logical and plausible arguments”; Kraepelin referred to the condition as “paranoia” and considered that hallucinations could not be present– unlike Bleuler, who considered them to be a possible feature. The criteria for delusional disorder have suffered several changes in the last centuries.

**Objectives:**

We aim to review the evolution of the criteria for delusional disorder across the editions of the Diagnostic and Statistical Manual of Mental Disorders (DSM).

**Methods:**

Review of DSM editions.

**Results:**

Criteria for the diagnosis of “paranoia” (DSM III) or “delusional disorder” (DSM III-IV.V) underwent several changes. In the first editions hallucinations could not be prominent (DSM-III-IIIR) and in the DSM IV, only tactile or olfactory hallucinations related to delusions could be present. In DSM-V hallucinations of other modalities related to the delusional theme can be present. Regarding delusional themes, the first edition of the DSM III regarded persecutory delusions only – which was changed in the DSM-III-R, with the inclusion of grandiose, jealous, erotomaniac, and somatic. Only in the DSM-V did the occurrence of bizarre delusions become possible in delusional disorder. Across the editions, there is a consensus about the absence of negative symptoms, absence of disorganized speech, and that the behavior is not odd aside from delusional content.

**Conclusions:**

The most debatable symptoms across centuries in the classification of delusional disorders were: presence of hallucinations, the nature of the delusional content, and inclusion of bizarre delusions.

**Disclosure:**

No significant relationships.

